# Sociodemographic inequalities in cardiovascular risk factors among adolescents from indigenous areas in Chiapas, Mexico

**DOI:** 10.1590/0102-311XEN024623

**Published:** 2023-11-13

**Authors:** Elena Flores-Guillén, Itandehui Castro-Quezada, César Antonio Irecta-Nájera, Pilar E. Núñez-Ortega, Roberto Solís-Hernández, Rosario García-Miranda, Paola Cruz-Cruz, Christian Medina-Gómez, Xariss M. Sánchez-Chino, Zendy Evelyn Olivo-Vidal, Miguel Cruz, Héctor Ochoa-Díaz-López

**Affiliations:** 1 El Colegio de la Frontera Sur, San Cristóbal de Las Casas, México.; 2 Universidad de Ciencias y Artes de Chiapas, Tuxtla Gutiérrez, México.; 3 Escuelas de Lenguas, Universidad Autónoma de Chiapas, San Cristóbal de Las Casas, México.; 4 Hospital de Especialidades, Centro Médico Nacional Siglo XXI, Ciudad de México, México.

**Keywords:** Heart Disease Risk Factors, Sociodemographic Factors, Adolescent, Indigenous Peoples, Factores de Riesgo de Enfermedad Cardiaca, Factores Sociodemográficos, Adolescente, Pueblos Indígenas, Fatores de Risco de Doenças Cardíacas, Fatores Sociodemográficos, Adolescente, Povos Indígenas

## Abstract

This study was aimed to determine the prevalence of cardiovascular risk factors among different sociodemographic groups of adolescents from indigenous communities in Chiapas, Mexico. A cross-sectional prevalence study was performed in urban and rural communities in the Tzotzil-Tzeltal and Selva regions of Chiapas. A sample of 253 adolescents was studied, of whom 48% were girls and 52% were boys. A descriptive analysis of quantitative variables was performed using measures of central tendency and dispersion. The prevalence of cardiovascular risk factors stratified by sex, geographical area, years of schooling, and ethnicity of the mothers was estimated. The prevalence of cardiovascular risk factors was analyzed in relation to the sociodemographic characteristics of the study population. Low HDL-c (51%) was the predominant cardiovascular risk factor. Girls had a higher prevalence of abdominal obesity, hypertriglyceridemia, and borderline total cholesterol than boys. High diastolic blood pressure was more prevalent in boys. Adolescents from urban areas had a higher prevalence of overweight/obesity and insulin resistance than adolescents from rural areas. The prevalence of overweight/obesity and abdominal obesity was higher in adolescents whose mothers had ≥ 7 years of schooling compared with adolescents with less educated mothers. Differences by maternal ethnicity also influenced the prevalence of insulin resistance. Among the main findings, this study associated sociodemographic and geographical inequalities with cardiovascular risk factors. Promoting a healthy lifestyle for this young population is absolutely necessary to prevent cardiovascular diseases in adulthood.

## Resumen

El objetivo de este estudio fue estimar la prevalencia de los factores de riesgo cardiovascular entre diferentes grupos sociodemográficos de adolescentes de comunidades indígenas de Chiapas, México. Se realizó un estudio transversal de prevalencia en comunidades urbanas y rurales de las regiones Tzotzil-Tzeltal y Selva, en Chiapas. Participó una muestra de 253 adolescentes, en la cual el 48% eran niñas y el 52% niños. Se realizó un análisis descriptivo de las variables cuantitativas utilizando medidas de tendencia central y dispersión. Se estimó la prevalencia de los factores de riesgo cardiovascular, estratificados por sexo, área geográfica, nivel de estudios y etnia de las madres. Se analizó la prevalencia de los factores de riesgo cardiovascular con relación a las características sociodemográficas de la población estudiada. El HDL-c bajo (51%) fue el factor de riesgo cardiovascular predominante. Se observó una mayor prevalencia de obesidad abdominal, hipertrigliceridemia y colesterol total en las niñas que en los niños. La alta presión arterial diastólica prevaleció en los niños. Los adolescentes del área urbana tuvieron una mayor prevalencia de sobrepeso/obesidad y resistencia a la insulina que los del área rural. La prevalencia de sobrepeso/obesidad y obesidad abdominal fue mayor en los adolescentes cuyas madres tenían nivel de estudios ≥ 7 años que aquellos cuyas madres tenían bajo nivel de estudios. Las diferencias en la etnicidad materna también influyeron en la prevalencia de resistencia a la insulina. Entre las principales conclusiones de este estudio, se destacan las desigualdades sociodemográficas y geográficas entre los factores de riesgo cardiovascular. La promoción de un estilo de vida saludable entre la población joven es lo indicado para prevenir las enfermedades cardiovasculares en la edad adulta.

## Resumo

O objetivo deste estudo foi determinar a prevalência de fatores de risco cardiovascular entre diferentes grupos sociodemográficos de adolescentes de comunidades indígenas em Chiapas, México. Foi realizado um estudo transversal de prevalência em comunidades urbanas e rurais das regiões de Tzotzil-Tzeltal e Selva de Chiapas. Foi estudada uma amostra de 253 adolescentes, sendo 48% meninas e 52% meninos. Foi realizada uma análise descritiva das variáveis quantitativas por meio de medidas de tendência central e dispersão. Foram estimadas as prevalências de fatores de risco cardiovascular, estratificadas por sexo, área geográfica, escolaridade e etnia das mães. A prevalência dos fatores de risco cardiovascular foi analisada em relação às características sociodemográficas da população estudada. O HDL-c baixo (51%) foi o fator de risco cardiovascular predominante. Prevalências mais elevadas de obesidade abdominal, hipertrigliceridemia e colesterol total limítrofe foram mais observadas em meninas do que em meninos. A pressão arterial diastólica elevada prevaleceu nos meninos. Adolescentes da área urbana apresentaram prevalências de sobrepeso/obesidade e resistência à insulina maiores do que os da área rural. A prevalência de sobrepeso/obesidade e obesidade abdominal foi maior nos adolescentes cujas mães possuíam escolaridade ≥ 7 anos do que naqueles indivíduos cujas mães tinham baixa escolaridade. As diferenças de etnia das mães também foram observadas na prevalência de resistência à insulina. Dentre as principais conclusões, foram encontradas, neste estudo, desigualdades sociodemográficas e geográficas entre fatores de risco cardiovascular. Promover estilos de vida saudáveis entre a população jovem é o ideal para prevenir doenças cardiovasculares na vida adulta.

## Introduction

In recent decades, interest in the study of health inequalities has grown. According to previous evidence, in Mexico, certain chronic and infectious diseases, such as malnutrition, tuberculosis ^1^, anemia, cervical cancer, metabolic syndrome, and diabetes ^2^, were more frequent among individuals of low socioeconomic status. In Mexico, cardiovascular diseases (CVD) are the main cause of mortality, representing an important public health problem. Evidence shows the association of different risk factors with CVD. Abnormal lipid metabolism during childhood ^3^ is a risk factor for atheromatous plaques during adulthood ^4^. Changes in other metabolic risk factors, such as serum glucose, insulin, C-reactive protein ^5^, blood pressure, and waist circumference, are also associated with an increased risk of CVD ^6^.

In Latin American countries such as Brazil, Chile, and Venezuela, obesity and dyslipidemia are highly prevalent ^7^. A study with 180 adolescents from Mexico City showed that 61% had hypertriglyceridemia and half of them had low concentrations of high-density lipoprotein cholesterol (HDL-c) ^8^. In the State of Morelos, a study with 869 adolescents found that 7% had metabolic syndrome, 68% hypertriglyceridemia, 17% low HDL-c, and 15% hyperglycemia ^9^. In the city of Tuxtla Gutiérrez, Chiapas, the prevalence of metabolic syndrome in adolescents was 16% ^10^. However, studies conducted in other countries have shown that cardiovascular risk can be influenced by socioeconomic status ^11^, ethnicity ^12^ ^,^ ^13^, and geographical area ^14^. In Mexico, few studies compare cardiovascular risk factors between indigenous peoples and the general population. A study performed with adolescents from urban and rural schools in Central Mexico showed a high prevalence of hypoalphalipoproteinemia, especially among boys from the rural area, where the main ethnic groups were the Mazahuas and the Otomi ^15^. Another study with adults in Chiapas showed that the mestizo population had a higher risk of CVD than the indigenous population ^16^. To date, the specialized literature presents little evidence on cardiovascular risk factors for adolescents in Chiapas. Thus, this study aims to measure the prevalence of cardiovascular risk factors and analyze their distribution among different population groups in Chiapas.

## Methods

### Study and sample design

This cross-sectional prevalence study was conducted with a sample of adolescents from a birth cohort study, born in three public hospitals in Chiapas in 2003. The sampling had two stages. In the first stage, the communities to which adolescents belong were clustered according to population size and geographical area (rural/urban). In the second stage, a systematic sampling of adolescents was performed with a randomized start-up ^17^.

### Study population

In total, 303 adolescents of both sexes, with a mean age of 14.1 years, from 14 municipalities in the Tzotzil-Tzeltal and Selva regions of Chiapas were selected ^17^. Of them, 50, without biochemical data, were excluded, obtaining a final sample of 253 adolescents.

### Survey information

Interviews were conducted with adolescents and their mothers or caregivers at their homes. A pre-coded and structured questionnaire was applied, covering the following information: sociodemographic data, non-pathological personal history, family medical history, anthropometric and clinical measurements. Blood samples were collected for biochemical analyses. Sociodemographic data included sex, age, geographical area (rural/urban), ethnicity, and years of schooling of adolescents and their mothers, and household assets. Family medical history included diseases of first- and second-degree relatives and non-pathological personal history (smoking and alcohol use). The anthropometric and clinical assessment considered weight, height, waist circumference, and blood pressure.

### Data collection procedures

Data were collected by a multidisciplinary fieldwork team (medicine, biology, clinical chemistry, and nutrition) ^17^. Weight was measured by electronic scales (accuracy ± 100g, Model UM081, Tanita Corporation, https://www.tanita.com/es/). Height was measured using stadiometers (accuracy ± 1mm, SECA, https://www.seca.com/de_de.html). Body mass index (BMI) was estimated by dividing weight by height squared and BMI z-score was calculated with AnthroPlus v. 1.0.4 (https://who-anthroplus.freedownloadscenter.com/windows/). As the main interest of this study is overweight/obesity as a risk factor, nutritional status was classified as normal weight [< 1 standard deviation (SD)] and overweight/obesity [BMI z-score ≥ 1 SD]. Waist circumference was measured by anthropometric tapes (accuracy ± 1mm, SECA). The cut-off point for abdominal obesity was ≥ 80cm in girls and ≥ 90cm in boys, according to the International Diabetes Federation ^18^.

Blood pressure was measured twice on the right mid-arm using a digital monitor (Model CH-453, Citizen, https://www.citizen-systems.co.jp/english/support/index.html) while the adolescent was sitting and after a five-minute rest. Systolic blood pressure (SBP) and diastolic blood pressure (DBP) were classified as normal (< 90th percentile) or high (≥ 90th percentile) ^19^, using the percentile tables of the US National Institutes of Health for age, sex, and height ^20^.

### Biochemical measurements

Blood samples were collected from the antecubital vein by venipuncture after a 12-hour fasting. Serum was obtained by centrifugation at 6,000 rpm for 10 minutes. Serum glucose, triglycerides, total cholesterol, HDL-c, and low-density lipoprotein cholesterol (LDL-c) were measured by an automated analyzer (Vitalab Selectra E, Vitalab Scientific, https://fr.bimedis.com/vitalab-selectra-e-m274310) using photometric enzymatic methods (Diasys Diagnostic System, https://www.diasys-diagnostics.com/). Insulin serum levels were quantified in an eclectic analyzer (Adaltis Diagnostics, https://www.adaltis.net/) using the immunoenzymatic method (Adaltis Diagnostics). High-sensitivity C-reactive protein (hs-CRP) was quantified by nephelometry (Genius, http://www.geniusmedica.net/) and brand reagents (Diasys Diagnostic System).

### Diagnostic criteria

### Metabolic changes

Glucose levels were classified as normal (< 100mg/dL) or abnormal blood glucose (≥ 100mg/dL) ^19^. The following cut-off points were established for serum lipid levels: triglycerides (acceptable < 90mg/dL, borderline 90-129mg/dL, and high ≥ 130mg/dL), total cholesterol (acceptable < 170mg/dL, borderline 170-199mg/dL, and high ≥ 200mg/dL), LDL-c (acceptable < 130mg/dL and high ≥ 130mg/dL) and c-HDL (acceptable > 40mg/dL and low < 40mg/dL) ^21^.

### Insulin resistance 

The homeostatic model to assess insulin resistance (HOMA-IR) was estimated by multiplying fasting blood glucose (mg/dL) by fasting insulin (µU/mL) divided by 22.5. The cut-off point used to determine insulin resistance was 2.97 ^22^.

### Cardiovascular risk

To discriminate cardiovascular risk by hs-CRP, the following cut-off points of the US Centers for Disease Control and Prevention (CDC) and the American Heart Association (AHA) were used: low risk hs-CRP < 1mg/dL, average risk 1-3mg/dL, and high risk > 3mg/dL ^23^.

### Metabolic syndrome

The diagnosis of metabolic syndrome was analyzed according to the criteria of the National Cholesterol Education Program Adult Treatment Panel III (NCEP-ATP III), previously used by Ford et al. ^19^. According to NCEP-ATP III definition, the diagnosis of metabolic syndrome must include three or more of the following factors (for both sexes): abdominal obesity (waist circumference ≥ 90th sex-specific percentile), triglycerides ≥ 110mg/dL, HDL-c ≤ 40mg/dL, SBP or DBP ≥ 90th percentile (age-, height-, and sex-specific), and glucose ≥ 110mg/dL ^24^.

### Statistical analysis

A descriptive analysis of quantitative variables was performed using measures of central tendency and dispersion. The prevalence of cardiovascular risk factors and 95% confidence intervals (95%CI) were estimated, stratified by sex, geographical area (rural/urban), years of schooling, and maternal ethnicity. The difference between variable distributions was obtained by Mann-Whitney U tests (for non-parametric variables) and independent samples t-tests (for parametric variables). The proportions of qualitative variables were analyzed using the Bonferroni correction. All analyses were performed in SPSS (https://www.ibm.com/).

### Ethical approval and participation consent

Before the interview, mothers or caregivers and adolescents signed an informed consent form. This study was approved by the Research Ethics Committee of the El Colegio de la Frontera Sur (CEI-O-076/16).

## Results

Of the 253 adolescents, 52% were boys and 75% lived in urban areas. Table 1 presents the sociodemographic characteristics of adolescents according to sex. Their mean age was 14 years and their mean years of schooling was 6.5 years. In total, 46% of mothers spoke an indigenous language and 56.4% had < 7 years of schooling. Of adolescents’ fathers, 30% had < 7 years of schooling and 53% spoke an indigenous language. Regarding household assets, 4% of participants had no running water, 32% cooked with charcoal/firewood, 12% had a latrine, septic tank, or none of them, 36% had no fridge, 13% had no television, 22% had no cell phone, and 80% had no computer. In total, 77% of adolescents ate red meat less than once per week and only 12% had medical insurance (Institute for Social Security and Services for State Workers - ISSSTE, Social Security Institute of Workers of the State of Chiapas - ISSSTECH, Mexican Social Security Institute - IMSS, Mexican Ministry of National Defense - SEDENA). We found differences in socioeconomic characteristics between the sexes in terms of having a fridge and cooking fuel (Table 1).

**Table 1:** Sociodemographic characteristics of the sample of adolescents by sex *.

Characteristics	Girls		Boys		Total		p-value
	n	Mean ± SD	n	Mean ± SD	n	Mean ± SD	
Sociodemographic							
Adolescents							
Age (years old)	122	14.1 ± 0.2	131	14.16 ± 0.3	253	14.15 ± 0.3	0.894 **
Schooling (years)	122	7.7 ± 1.1	131	7.7 ± 1.0	253	7.7 ± 1.0	0.559 ***
Geographical area of residence [%]							
Urban	95	77.9	94	71.8	189	74.7	0.311 ^#^
Rural	27	22.1	37	28.2	64	25.3	
Mother’s schooling (years) [%]	118	6.8 ± 4.8	125	6.3 ± 4.4	243	6.5 ± 4.5	0.609 ***
< 7	62	52.5	75	60.0	137	56.4	0.248 ^#^
≥ 7	56	47.5	50	40.0	106	43.6	
The mother speaks an indigenous language [%]							
No	69	58.5	62	49.6	131	53.9	0.165 ^#^
Yes	49	41.5	63	50.4	112	46.1	
Father’s schooling (years) [%]	100	7.9 ± 4.9	114	7.4 ± 4.3	214	7.6 ± 4.5	0.799 **
< 7	51	51.0	62	54.4	113	52.8	0.681 ^#^
≥ 7	49	49.0	52	45.6	101	47.2	
The father speaks an indigenous language [%]							
No	55	55,0	58	50.9	113	52.8	0.584 ^#^
Yes	45	45.0	56	49.1	101	47.2	
Household assets							
Television [%]							
No	15	12.4	17	13.1	32	12.7	0.872 ^#^
Yes	106	87.6	113	86.9	219	87.3	
Fridge [%]							
No	36	29.8	54	41.5	90	35.9	0.052 ^#^
Yes	85	70.2	76	58.5	161	64.1	
Microwave oven [%]							
No	85	70.2	96	73.8	181	72.1	0.574 ^#^
Yes	36	29.8	34	26.2	70	27.9	
Cell phone (head of household) [%]							
No	25	20.7	30	23.1	55	21.9	0.651 ^#^
Yes	96	79.3	100	76.9	196	78.1	
Computer [%]							
No	96	79.3	106	81.5	202	80.5	0.750 ^#^
Yes	25	20.7	24	18.5	49	19.5	
Cooking fuel [%]							
Gas	92	76.0	79	60.8	171	68.1	0.010 ^#^
Exclusively charcoal/Firewood	29	24.0	51	39.2	80	31.9	
Bathroom [%]							
Bathroom (with sewage)	110	90.9	110	84.6	220	87.6	0.130 ^#^
Latrine, septic tank, or none of them	11	9.1	20	15.4	31	12.4	
Running water in the house [%]							
Yes	117	96.7	124	95.4	241	96.0	0.596 ^#^
No	4	3.3	6	4.6	10	4.0	
Medical insurance [%]							
ISSSTE/ISSTECH/IMSS/SEDENA	13	10.7	16	12.3	29	11.6	0.699 ^#^
Seguro Popular/Other	108	89.3	114	87.7	222	88.4	
Meat intake (per week) [%]							
1 or more	29	24.0	28	21.5	57	22.7	0.646 ^#^
< 1	92	76.0	102	78.5	194	77.3	

ISSSTE: Institute for Social Security and Services for State Workers; ISSSTECH: Social Security Institute of Workers of the State of Chiapas; IMSS: Mexican Social Security Institute; SD: standard deviation; SEDENA: Mexican Ministry of National Defense.

* Data are presented as mean ± SD for quantitative variables and percentages for qualitative variables;

** Student’s t-test;

*** Mann-Whitney U test;

^#^ Fisher’s exact test.

Table 2 shows the adolescents’ family medical history and non-pathological personal history. Around 45% of adolescents had relatives with chronic diseases. The most commonly reported diseases were type 2 diabetes, hypertension, and CVD. In total, 68% followed the Mexican vaccination schedule, boys less than girls, and 54% were not exclusively breastfed in their first six months of life. Among the adolescents, 20% consumed alcohol occasionally, boys less than girls, and 16% had smoked at least once in their lives.

**Table 2:** Family medical history and non-pathological personal history of adolescents *.

Parameter	Girls (n = 121)			Boys (n = 130)			Total (n = 251)			p-value **
	n	%	95%CI	n	%	95%CI	n	%	95%CI	
Family medical history *										
Obesity										
Yes	27	22.0	16.0-30.3	28	22.0	15.0-29.0	55	22.0	17.0-27.0	0.882
No	94	78.0	70.0-84.4	102	79.0	71.0-85.0	196	78.0	73.0-83.0	
Hypertension										
Yes	55	46.0	37.0-54.3	57	44.0	36.0-52.0	112	45.0	39.0-51.0	0.798
No	66	55.0	46.0-63.2	73	56.0	48.0-65.0	139	55.0	49.0-61.0	
Type 2 diabetes										
Yes	55	46.0	37.0-54.3	57	44.0	36.0-52.0	112	45.0	39.0-51.0	0.798
No	66	55.0	46.0-63.2	73	56.0	48.0-65.0	139	55.0	49.0-61.0	
Cardiovascular diseases										
Yes	54	45.0	36.0-53.5	57	44.0	36.0-52.0	111	44.0	38.0-50.0	0.901
No	67	55.0	47.0-64.0	73	56.0	48.0-65.0	140	56.0	50.0-62.0	
Non-pathological personal history										
Complete vaccination schedule										
Yes	82	73.0	65.0-80.7	75	64.0	55.0-72.0	157	68.0	62.0-74.0	0.116
No	30	27.0	19.0-35.5	43	36.0	28.0-45.0	73	32.0	26.0-38.0	
Exclusive breastfeeding ***										
Yes	51	46.0	37.0-55.7	54	46.0	37.0-55.0	105	46.0	40.0-53.0	0.975
No	59	54.0	44.0-62.8	63	54.0	44.0-63.0	122	54.0	47.0-60.0	
Have smoked										
Yes	18	15.0	9.4-22.0	22	17.0	11.0-24.0	40	16.0	12.0-21.0	0.677
No	103	85.0	78.0-90.1	109	83.0	76.0-89.0	212	84.0	79.0-88.0	
Have consumed alcoholic beverages										
Yes	30	25.0	18.0-32.8	20	15.0	9.9-22.0	50	20.0	15.0-25.0	0.063
No	90	74.0	66.0-81.5	111	85.0	78.0-90.0	202	80.0	75.0-85.0	

95%CI: 95% confidence interval.

* Family members of first- and second-degree consanguinity (parents and grandparents);

** Chi-squared test;

*** During the first six months.

Table 3 shows the prevalence of cardiovascular risk factors by sex. The most prevalent risk factor was low HDL-c (51%), followed by borderline triglycerides (35%), overweight/obesity (29%), and hypertriglyceridemia (28%). A small percentage of the population had high LDL-c levels (1%), abnormal fasting blood glucose (3%), and high total cholesterol (4%). A significantly higher proportion of girls had abdominal obesity (p < 0.001), borderline total cholesterol (p = 0.002), and high triglycerides (p = 0.021) as compared with boys. Moreover, the prevalence of overweight/obesity was 45% higher in girls than in boys. However, boys presented greater prevalence of high DBP and low HDL-c levels.

**Table 3:** Prevalence of cardiovascular risk factors in adolescents according to sex.

Cardiovascular risk factors	Girls (n = 122)		Boys (n = 131)		Total (n = 253)		p-value *
	%	95%CI	%	95%CI	%	95%CI	
Anthropometric measurements							
Overweight/Obesity (BMI > 1 z-score)	34.4	26.4-43.1	23.7	17.0-31-5	28.9	23.5-34.7	0.059
Abdominal obesity (≥ 80cm in girls and ≥ 90cm in boys)	24.6	17.6-32.8	5.3	2.4-10.2	14.6	10.7-19.4	< 0.001
Clinical measurements							
High SBP (≥ 90th percentile)	15.6	10.0-22.8	23.7	17.0-31.5	19.8	15.2-25.0	0.106
High DBP (≥ 90th percentile)	7.4	3.7-13.0	22.9	16.3-30.9	15.4	11.4-20.2	0.001
Biochemical measurements							
Fasting blood glucose (≥ 100mg/dL)	1.6	0.3-5.2	4.6	1.9-9.2	3.2	1.5-5.9	0.182
Borderline triglycerides (90-129mg/dL)	32.8	24.9-41.4	36.6	28.8-45.1	34.8	29.1-40.8	0.520
High triglycerides (≥ 130mg/dL)	35.2	27.2-44.0	22.1	15.7-29.8	28.5	23.2-34.2	0.021
Borderline total cholesterol (170-199mg/dL)	14.8	9.3-21.8	3.8	1.5-8.2	9.1	6.0-13.1	0.002
High total cholesterol (≥ 200mg/dL)	5.7	2.6-10.9	2.3	0.6-6.0	4.0	2.0-6.9	0.160
High LDL-c (≥ 130mg/dL)	1.6	0.3-5.2	0.0	**	0.8	0.2-2.5	**
Low HDL-c (< 40mg/dL)	44.3	35.7-53.1	56.5	47.9-64.8	50.6	44.5-56.7	0.052
HOMA-IR (≥ 2.97 units)	28.3	20.9-36.8	19.8	13.7-27.3	23.9	18.9-29.5	0.115
High risk hs-CRP (> 3mg/dL)	14.0	8.7-21.1	9.9	5.7-15.9	11.9	8.3-16.3	0.312

95%CI: 95% confidence interval; BMI: body mass index; DBP: diastolic blood pressure; HDL-c: high-density lipoprotein cholesterol; HOMA-IR: homeostatic model to assess insulin resistance; hs-CRP: high-sensitivity C-reactive protein; LDL-c: low-density lipoprotein cholesterol; SBP: systolic blood pressure.

* Comparison of proportions using the Bonferroni correction;

** Not estimable, a column with a proportion equal to zero.

Table 4 presents the prevalence of cardiovascular risk factors according to geographical area (urban/rural). In urban areas, the prevalence of overweight/obesity (33%) and insulin resistance (28%) was significantly higher compared with rural areas. Moreover, we found a borderline significant difference in abdominal obesity among adolescents living in urban areas (16.9%) compared with rural areas (7.8%).

**Table 4:** Prevalence of cardiovascular risk factors in adolescents according to geographical area.

Cardiovascular risk factors	Urban (n = 189)		Rural (n = 64)		Total (n = 253)		p-value *
	%	95%CI	%	95%CI	%	95%CI	
Anthropometric measurements							
Overweight/Obesity (BMI > 1 z-score)	32.8	26.4-39.7	17.2	9.5-27.8	28.9	23.5-34.7	0.017
Abdominal obesity (≥ 80cm in girls and ≥ 90cm in boys)	16.9	12.1-22.8	7.8	3.0-16.3	14.6	10.7-19.4	0.074
Clinical measurements							
High SBP (≥ 90th percentile)	20.1	14.9-26.2	18.8	10.7-29.6	19.8	15.2-25.0	0.814
High DBP (≥ 90th percentile)	14.3	9.9-19.8	18.8	10.7-29.6	15.4	11.4-20.2	0.393
Biochemical measurements							
Fasting blood glucose (≥ 100mg/dL)	3.7	1.7-7.1	1.6	0.2-7.1	3.2	1.5-5.9	0.398
Borderline triglycerides (90-129mg/dL)	37.0	30.4-44.1	28.1	18.3-39.9	34.8	29.1-40.8	0.196
High triglycerides (≥ 130mg/dL)	27.5	21.5-34.2	31.3	20.9-43.2	28.5	23.2-34.2	0.567
Borderline total cholesterol (170-199mg/dL)	9.5	6.0-14.3	7.8	3.0-16.3	9.1	6.0-13.1	0.681
High total cholesterol (≥ 200mg/dL)	4.2	2.0-7.8	3.1	0.7-9.6	4.0	2.0-6.9	0.694
High LDL-c (≥ 130mg/dL)	1.1	0.2-3.4	0.0	**	0.8	0.2-2.5	**
Low HDL-c (< 40mg/dL)	22.2	16.728.5	21.9	13.1-33.1	22.1	17.4-27.5	0.954
HOMA-IR (≥ 2.97 units)	27.8	21.8-34.5	12.5	6.1-22.2	23.9	18.9-29.5	0.013
High risk hs-CRP (> 3mg/dL)	12.8	8.6-18.1	9.4	4.0-18.3	11.9	8.3-16.3	0.469

95%CI: 95% confidence interval; BMI: body mass index; DBP: diastolic blood pressure; HDL-c: high-density lipoprotein cholesterol; HOMA-IR: homeostatic model to assess insulin resistance; hs-CRP: high-sensitivity C-reactive protein; LDL-c: low-density lipoprotein cholesterol; SBP: systolic blood pressure.

* Comparison of proportions using the Bonferroni correction;

** Not estimable, a column with a proportion equal to zero.

Regarding maternal schooling (Table 5), this study observed a significantly higher prevalence of overweight/obesity and abdominal obesity among adolescents whose mothers had ≥ 7 years of schooling. On the other hand, adolescents with mothers with < 7 years of schooling had a higher prevalence of low HDL-c. Regarding maternal ethnicity (Table 6), adolescents whose mothers did not speak an indigenous language had a significantly higher prevalence of insulin resistance compared with adolescents whose mothers spoke an indigenous language (HOMA-IR ≥ 2.97 units). Moreover, the prevalence of low HDL-c was higher among adolescents whose mothers spoke an indigenous language (56.3%) compared with adolescents whose mothers spoke only Spanish (45%).

**Table 5:** Prevalence of cardiovascular risk factors in adolescents according to their mother’s schooling.

Cardiovascular risk factors	Mother’s schooling (years)						p-value *
	< 7 (n = 137)		≥ 7 (n = 106)		Total (n = 243)		
	%	95%CI	%	95%CI	%	95%CI	
Anthropometric measurements							
Overweight/Obesity (BMI > 1 z-score)	22.6	16.2-30.2	37.7	28.9-47.2	29.2	23.8-35.2	0.010
Abdominal obesity (≥ 80cm in girls and ≥ 90cm in boys)	9.5	5.4-15.2	22.6	15.5-31.3	15.2	11.1-20.1	0.005
Clinical measurements							
High SBP (≥ 90th percentile)	18.2	12.5-25.3	20.8	13.9-29.3	19.3	14.8-24.7	0.624
High DBP (≥ 90th percentile)	16.1	10.6-22.9	14.2	8.5-21.7	15.2	11.1-20.1	0.681
Biochemical measurements							
Fasting blood glucose (≥ 100mg/dL)	2.9	1.0-6.8	2.8	0.8-7.4	2.9	1.3-5.6	0.967
Borderline triglycerides (90-129mg/dL)	29.9	22.7-38.0	39.6	30.7-49.1	34.2	28.4-40.3	0.209
High triglycerides (≥ 130mg/dL)	29.2	22.1-37.2	27.4	19.6-36.4	28.4	23.0-34.3	0.114
Borderline total cholesterol (170-199mg/dL)	8.0	4.3-13.5	10.4	5.6-17.2	9.1	5.9-13.1	0.527
High total cholesterol (≥ 200mg/dL)	3.6	1.4-7.8	4.7	1.8-10.0	4.1	2.1-7.2	0.678
High LDL-c (≥ 130mg/dL)	0.7	0.1-3.4	0.9	0.1-4.3	0.8	0.2-2.6	0.855
Low HDL-c (< 40mg/dL)	58.4	50.0-66.4	39.6	30.7-49.1	50.2	43.9-56.5	0.004
HOMA-IR (≥ 2.97 units)	22.1	15.7-29.6	26.7	18.9-35.7	24.1	19.0-29.8	0.407
High risk hs-CRP (> 3mg/dL)	13.9	8.9-20.4	9.4	5.0-16.1	11.9	8.3-16.5	0.290

95%CI: 95% confidence interval; BMI: body mass index; DBP: diastolic blood pressure; HDL-c: high-density lipoprotein cholesterol; HOMA-IR: homeostatic model to assess insulin resistance; hs-CRP: high-sensitivity C-reactive protein; LDL-c: low-density lipoprotein cholesterol; SBP: systolic blood pressure.

* Comparison of proportions using the Bonferroni correction.

**Table 6:** Prevalence of cardiovascular risk factors in adolescents according to their mother’s language.

Cardiovascular risk factors	Mother’s language (ethnicity)						p-value *
	Spanish (n = 131)		Indigenous (n = 112)		Total (n = 243)		
	%	95%CI	%	95%CI	%	95%CI	
Anthropometric measurements							
Overweight/obesity (BMI > 1 z-score)	30.5	23.1-38.8	27.7	20.0-36.5	29.2	23.8-35.2	0.626
Abdominal obesity (≥ 80cm in girls and ≥ 90cm in boys)	14.5	9.3-21.3	16.1	10.2-23.7	15.2	11.1-20.1	0.735
Clinical measurements							
High SBP (≥ 90th percentile)	16.8	11.2-23.9	22.3	15.4-30.7	19.3	14.8-24.7	0.291
High DBP (≥ 90th percentile)	10.7	6.3	20.5	13.9-28.7	15.2	11.1-20.1	0.288
Biochemical measurements							
Fasting blood glucose (≥ 100mg/dL)	2.3	0.6-6.0	3.6	1.2-8.3	2.9	1.3-5.6	0.552
Borderline triglycerides (90-129mg/dL)	34.4	26.6-42.8	33.9	25.7-43.0	34.2	28.4-40.3	0.945
High triglycerides (≥ 130mg/dL)	27.5	20.4-35.6	29.5	21.6-38.3	28.4	23.0-34.3	0.733
Borderline total cholesterol (170-199mg/dL)	12.2	7.4-18.6	5.4	2.3-10.7	9.1	5.9-13.1	0.063
High total cholesterol (≥ 200mg/dL)	3.8	1.5-8.2	4.5	1.7-9.5	4.1	2.1-7.2	0.800
High LDL-c (≥ 130mg/dL)	1.5	0.3-4.8	0.0	**	0.8	0.2-2.6	0.800
Low HDL-c (< 40mg/dL)	45.0	36.7-53.6	56.3	47.0-65.2	50.2	43.9-56.5	0.081
HOMA-IR (≥ 2.97 units)	30.2	22.8-38.5	17.0	10.9-24.7	24.1	19.0-29.8	0.016
High risk hs-CRP (> 3mg/dL)	11.5	6.8-17.7	12.5	7.3-19.6	11.9	8.3-16.5	0.801

95%CI: 95% confidence interval; BMI: body mass index; DBP: diastolic blood pressure; HDL-c: high-density lipoprotein cholesterol; HOMA-IR: homeostatic model to assess insulin resistance; hs-CRP: high-sensitivity C-reactive protein; LDL-c: low-density lipoprotein cholesterol; SBP: systolic blood pressure.

* Proportion comparison test (see pairwise comparison within a row of each variable using the Bonferroni correction);

** Not estimable, a column with a proportion equal to zero.

Table 7 shows the prevalence of metabolic syndrome defined by the NCEP-ATP III criteria, according to different populations. According to the NCEP-ATP, the prevalence of metabolic syndrome was 14.6%. This prevalence was higher in girls, urban areas, and adolescents with mothers who spoke an indigenous language and had ≥ 7 years of schooling.

**Table 7:** Prevalence of metabolic syndrome among adolescents from different sociodemographic groups and geographical areas.

Variables	Without metabolic syndrome	With metabolic syndrome *	p-value *** *
	%	%	
	n = 216	n = 37	* *
Overall sample	85.4	14.6	
Sex			
Girls	82.8	17.2	0.289
Boys	87.8	12.2	
Geographical area			
Urban	84.1	15.9	0.416
Rural	89.1	10.9	
	n = 207	n = 36	
Mother’s schooling (years) ***			
< 7	87.6	12.4	0.275
≥ 7	82.1	17.9	
Mother’s language (ethnicity) ***			
Spanish	87.8	12.2	0.277
Indigenous (Mayan)	82.1	17.9	

* National Cholesterol Education Program Adult Treatment Panel III (NCEP ATP III) criteria;

** Fisher’s exact test;

*** n = 243 due to missing values.

## Discussion

This study found a high prevalence of cardiovascular risk factors, such as overweight/obesity, low HDL-c, high triglycerides, and insulin resistance, among adolescents from 58 mestizo and indigenous communities in two regions of Chiapas. The prevalence of overweight/obesity in the study population was 29%, higher in urban areas (32.8%) than in rural areas (17.2%). Similarly, the *Mexican National Survey of Health and Nutrition* (ENSANUT 2016) for adolescents aged 12 to 19 years showed that the prevalence of overweight/obesity in urban areas was slightly higher (36.7%) than in rural areas (35%). The high prevalence of overweight/obesity found in this age group in our study is remarkable. This finding may be the result of the nutritional transition and lifestyle changes observed over the last decades, especially in urban areas. In these areas, the Mexican population has become sedentary ^2^ and has been exposed to Westernized dietary patterns, characterized by energy-dense, nutrient-poor foods and sugar-sweetened beverages ^25^. Moreover, the population in this study had a double burden of malnutrition, as in a previous study by the same authors, with a high prevalence of stunting among adolescents in rural areas ^17^. The prevalence of overweight/obesity and abdominal obesity was higher in adolescents whose mothers had ≥ 7 years of schooling. A study with schoolchildren in the state of Guerrero, Mexico, found that maternal schooling increased the likelihood of overweight among their children ^26^. Another study conducted in three Mexican cities (Tijuana, Tuxtla Gutiérrez, and Reynosa) also showed that among children from low-income families whose mothers had higher schooling levels, the prevalence of overweight/obesity was higher ^27^.

In this study, the prevalence of abdominal obesity was 14.6%. We found differences by sex, as girls had the highest prevalence of abdominal obesity (24.6%). The relationship between abdominal obesity and sex in adolescents is still controversial. For example, previous studies in Spain ^28^ and Brazil ^29^ showed a higher prevalence of abdominal obesity in boys than in girls. This may be due to the difference in total body fat between the sexes, considering the higher percentage of body fat in girls and the redistribution of fat from the extremities to the trunk. This distribution differs between the sexes, since changes in body fat are associated with estrogen and testosterone levels ^30^. Disparities between the sexes can also be attributed to social and cultural differences. In Mexico, the prevalence of overweight in girls increased from 23.7% in 2012 to 26.4% in 2016, while in boys, the prevalence remained unchanged ^31^. In Chiapas, the prevalence of overweight/obesity in girls also increased from 30.7% in 2006 to 32.1% in 2012 ^32^.

Regarding blood pressure, in our study, the proportion of boys with DBP ≥ 90th percentile was higher than the proportion of girls. A previous study with adolescents in the United States also showed that, after the onset of puberty, boys had higher blood pressure than girls of the same age ^33^. The mechanisms responsible for the differences in blood pressure during adolescence between the sexes are still unclear. Evidence shows that androgens, such as testosterone, may play an important role in regulating blood pressure in sex-related differences ^34^, since testosterone levels cause endothelial dysfunction ^35^.

Our findings for blood lipids, stratified by sex, showed that triglycerides and borderline total cholesterol were higher in girls than in boys. These differences by sex can be explained by the different hormonal activity during adolescence ^36^. The girls in our study had higher waist circumference values, which may explain their higher triglycerides levels. The most prevalent risk factor found in rural areas was high triglycerides levels, although this difference compared with urban areas was not statistically significant. This result is in line with a study with adolescents from Mexico City and a rural community in Central Mexico, where 45% of the population is predominantly Mazahua and Otomi ^37^. This finding can be partly explained by dietary intake. Low-fat and high-carbohydrate diets increase triglyceride levels ^38^. In our study, the mean carbohydrate intake was 356g per day in rural areas and 322g in urban areas. These values are higher than the average for Mexican adolescents in rural areas (285g per day for girls and 326g for boys). Moreover, 69% of girls and 61% of boys in rural areas regularly consume an excess of added sugars ^39^.

The prevalence of borderline total cholesterol was higher in girls (14.8%) than in boys (3.8%), but we found no differences by geographic area. A study reports that total cholesterol levels decrease in boys in early puberty, but increase again as they approach adulthood. In contrast, total cholesterol levels increase during puberty in girls ^40^. However, as we did not measure Tanner stages, we could not differentiate sexual maturation, which could influence our results.

Regarding HDL-c levels, we found a higher prevalence of low HDL-c in adolescents whose mothers had < 7 years of schooling compared with adolescents with mothers with ≥ 7 years of schooling. In line with our results, a previous study showed a trend towards a higher prevalence of low HDL-c in Mexican adults with lower socioeconomic status and schooling level ^41^. Moreover, we found a borderline significant difference in the prevalence of low HDL-c. Low HDL-c was higher among adolescents whose mothers spoke an indigenous language compared with adolescents whose mothers spoke only Spanish. Ethnic disparities in HDL-c levels may result from the presence and interaction of multiple components, such as genotype, body composition, lifestyle habits, and perceptions ^42^. For example, regarding genotype, the C230 variant of ATP-binding cassette transporter A1 (ABCA1) was associated with an increased risk of hypoalphalipoproteinemia in Mexican children ^43^ and adults ^44^. Among the Mayan population, the frequency of the C230 allelic variant (0.288) was higher than in the Mexican mestizo population (0.109) ^45^. The presence of this allelic variant translates directly into a 30% reduction in the ABCA1 transporter, which is heavily involved in HDL biogenesis ^46^. However, we highlight that the population-attributable risk of hypoalphalipoproteinemia from the C230 allele of ABCA1 in Mexico is around 12% ^44^. Thus, ethnic disparities in low HDL-c levels should not be attributed only to genetic factors, since other factors may be involved, such as behavioral (poor diet), cultural, and linguistic barriers, socioeconomic disparities ^47^, or unequal access to health care and facilities ^12^.

The prevalence of insulin resistance in this study was considerably high (24%). We also found that insulin resistance in urban areas was higher than in rural areas. This finding is in line with previous studies, in which the urban environment was an independent predictor of insulin resistance ^48^. Evidence shows that insulin resistance is associated with lipid changes in early life and with overweight/obesity ^49^. Thus, the interaction between the high prevalence of obesity and changes in lipid metabolism in our sample may also explain our results. Moreover, the prevalence of insulin resistance was higher in adolescents whose mothers were not indigenous. In Chiapas, most rural population is indigenous ^50^. We found no differences in HOMA-IR between the sexes and regarding maternal schooling.

The analysis of hs-CRP showed that 25% of adolescents had moderate and high cardiovascular risk according to CDC and AHA, with higher hs-CRP levels in girls, urban areas, and adolescents with less educated mothers, despite the lack of statistically significant differences. Similar to our results, a study with 418 Mexican adolescents from the city of Guadalajara showed no differences in cardiovascular risk regarding hs-CRP between the sexes ^51^.

Regarding metabolic syndrome in the study population, results showed a high prevalence (14.6%) according to the NCEP-ATP III criteria. Other studies conducted in Mexico showed a different prevalence: 8.8% at national level ^52^, 6.7% in Morelos ^9^, and 20% in Campeche ^53^, which has the highest prevalence of diabetes and hypertension in the country.

The nutritional transition in the population of Chiapas may be among the possible explanations for the situation found in our study ^54^. In recent decades, dietary patterns have shifted from a traditional plant-based diet to a Westernized eating style consisting of a high fat and refined carbohydrate intake ^55^. ENSANUT 2016 showed that adolescents habitually consumed food groups not recommended for daily consumption: 84% habitually consumed sugary drinks, 59% snacks, sweets, and desserts, and 50% sweetened cereals ^31^.

Sedentary behavior among adolescents has also been associated with insulin resistance, lipid changes, and higher inflammatory states ^56^. The adolescents studied spent 10 hours per day sitting (nine hours for the rural population) and only one hour and 40 minutes per week exercising or practicing sports, without differences between the sexes.

Socioeconomic factors and mechanisms such as different access to food, schooling level, health services, information, income, occupation, and empowerment can explain the disparities found between populations and geographical areas. One of the main strengths of this study is that it documents sociodemographic inequalities in cardiovascular risk factors among adolescents from marginalized Mayan communities in Chiapas, which have been little studied previously. This allowed us to identify vulnerable groups in order to develop future, precise interventions to modify their cardiovascular risk profiles. Every individual of the sample studied received their results in private, along with nutritional counseling.

This study with adolescents from marginalized indigenous areas in Chiapas showed sociodemographic and geographical disparities in cardiovascular risk factors. Girls had a higher prevalence of risk factors. We found a differential pattern of risk factors regarding geographical area, as the urban population was more vulnerable to overweight/obesity, while the rural population was more vulnerable to low HDL-c levels.

Based on these results, we recommend the creation of effective and targeted public health policies to improve the well-being of adolescents from marginalized indigenous areas: (1) monitoring nutritional status and promoting healthy lifestyles; (2) preventing and controlling the increasing prevalence of cardiovascular risk factors in the study population; (3) creating social and economic interventions to improve living conditions and prevent chronic diseases in adulthood; (4) continuing health surveillance in the studied population; (5) implementing culturally adapted models to bring health care to rural areas; and (6) conducting further studies on the genetic determinants of CVD in ethnic minorities.
